# Generalizability of Soft Sensors for Bioprocesses through Similarity Analysis and Phase-Dependent Recalibration

**DOI:** 10.3390/s23042178

**Published:** 2023-02-15

**Authors:** Manuel Siegl, Manuel Kämpf, Dominik Geier, Björn Andreeßen, Sebastian Max, Michael Zavrel, Thomas Becker

**Affiliations:** 1 Chair of Brewing and Beverage Technology, School of Life Sciences, Technical University of Munich, 85354 Freising, Germany; 2 Clariant Produkte (Deutschland) GmbH, 82152 Planegg, Germany; 3 Professorship for Bioprocess Engineering, Technical University of Munich, Campus Straubing, 94315 Straubing, Germany

**Keywords:** adaptive modeling, automatic recalibration, bioprocess, generalization, soft sensor

## Abstract

A soft sensor concept is typically developed and calibrated for individual bioprocesses in a time-consuming manual procedure. Following that, the prediction performance of these soft sensors degrades over time, due to changes in raw materials, biological variability, and modified process strategies. Through automatic adaptation and recalibration, adaptive soft sensor concepts have the potential to generalize soft sensor principles and make them applicable across bioprocesses. In this study, a new generalized adaptation algorithm for soft sensors is developed to provide phase-dependent recalibration of soft sensors based on multiway principal component analysis, a similarity analysis, and robust, generalist phase detection in multiphase bioprocesses. This generalist soft sensor concept was evaluated in two multiphase bioprocesses with various target values, media, and microorganisms. Consequently, the soft sensor concept was tested for biomass prediction in a *Pichia pastoris* process, and biomass and protein prediction in a *Bacillus subtilis* process, where the process characteristics (cultivation media and cultivation strategy) were varied. High prediction performance was demonstrated for *P. pastoris* processes (relative error = 6.9%) as well as *B. subtilis* processes in two different media during batch and fed-batch phases (relative errors in optimized high-performance medium: biomass prediction = 12.2%, protein prediction = 7.2%; relative errors in standard medium: biomass prediction = 12.8%, protein prediction = 8.8%).

## 1. Introduction

Because of technical or economic constraints, online hardware sensors are still often insufficient for monitoring complex bioprocesses with regard to their decisive biological key parameters. Soft(ware) sensors can be used to close this gap. To predict the target variables, a combination of mathematical models and existing hardware sensors is applied [1].

The partial least squares regression (PLSR) method is a popular way to build a data-driven soft sensor model. Using this method, a linear model is calibrated with an additional dimensionality reduction based on the relationships between hardware sensor readings and one or more target variables. This technique has been used to successfully develop data-driven soft sensor models in bioprocesses [2]. When combined with process knowledge, such as a carbon mass balance as input, these models performed particularly well as hybrid soft sensor models [3]. Nonetheless, a soft sensor is typically created manually for each bioprocess and is, thus, time-consuming. Typically, the automated application of soft sensor concepts across bioprocesses does not occur. Furthermore, the prediction performance of soft sensors often degrades significantly due to changing raw materials, modified process strategies, and biological variability. These issues are a significant barrier to their use in industry [4]. In particular, manual recalibration of soft sensors is often not executed due to a lack of qualified personnel. An automatic generalist recalibration approach can provide a solution.

The regular recalibration of the soft sensor model is a common method for adjusting the soft sensor. Previously, this was mostly performed manually from time to time, but in more recent studies, it is now partially automated and also known as just-in-time learning [5,6,7,8,9]. Differences in automatic recalibration are primarily due to the type of historical data selection. On the one hand, continuous recalibration of temporally matching sections from chronologically preceding data is possible [6]. The recalibration can, therefore, be performed within the current process in a moving time window based on chronologically corresponding data sets, and previous recalibrations can be gradually removed from the prediction model by forgetting factors [8]. On the other hand, a selection of historical data based on similarity criteria can be performed. This approach is not only suitable for slow and constant changes, but also for sudden changes, such as new raw materials. Thereby, historical data are selected for recalibration, in which these changes or similar changes occurred earlier [10]. Consequently, the selection is based on the similarity of the online process variables between the historical data [6,9] and the current process. As long as the correlations between the variables are constant, selecting historical data at the level of entire process data sets leads to better prediction performance than selecting individual reference points [7]. Multiway principal component analysis (MPCA), a similarity criterion, and clustering can be used to implement the selection. The MPCA technique folds the data pool to form a two-dimensional matrix, succeeded by a principal component analysis to concentrate the data’s information into higher-level variables [11]. According to the MPCA, data sets that match the current process can be selected from the data pool using a similarity criterion. One method for achieving this selection is to compute the Euclidean distances between historical data sets and the current process, followed by identifying nearest neighbors [6]. Other methods, such as the Mahalanobis distance, which additionally considers covariances of the process variables, can be used to determine similarity. Saptoro [10] gave a good overview of these methods.

Bioprocesses are usually multiphase processes, which means that correlations between variables typically change phase by phase throughout the process [3]. For the selection of historical data, a generalist soft sensor concept must consider these phase changes. This necessitates the inclusion of phase detection in the generalist soft sensor concept. Yao and Gao [12] gathered various phase detection methods and classified them into two groups. The first is based on expert knowledge, and the second is based solely on data-driven approaches. They depict multivariate rules [13] and the definition of landmarks in indicator variables [14,15] as examples of knowledge-driven methods. Data-driven methods are described, such as the analysis of local correlations [16] or approaches based on the explained variance of principal components for phase detection [17]. The use of data-driven phase detection methods in particular promises good transferability to multiple bioprocesses without the need for process knowledge. However, temporally faulty phase detection, also known as burrs, can occur, especially in data-driven approaches. Wang et al. [18] built a phase detection algorithm on such a data-driven concept and enhanced it with burr compensation.

Soft sensor concepts with automatic recalibration have traditionally been used primarily in the chemical and petroleum processing industries. Their application in biotechnological and pharmaceutical processes is currently limited [10] due to the more challenging processes involved. This technology, particularly when combined with phase detection algorithms, has only been described in a few publications, for example, to determine the penicillin concentration in a simulated bioprocess [19]. The broader application of different bioprocesses in a generalist concept in the biotechnology industry, as well as the implementation of robust data-driven phase detection methods that exclude burrs, is still an open issue.

A generalist soft sensor concept for multiphase bioprocesses is presented in this study. This novel concept provides soft sensors that automatically predict assigned target variables in various bioprocesses. MPCA, Euclidean distance, and a k-nearest neighbor algorithm are used to select historical data for automatic soft sensor recalibration. Furthermore, the selected historical data sets are divided using a phase detection algorithm with burrs compensation inspired by Wang et al. [18]. For the current process phase, a soft sensor model was then calibrated. Therefore, additional input variables were calculated from hardware sensor readings, including the carbon dioxide evolution rate (CER), the oxygen uptake rate (OUR), and the cumulative CER and OUR. Finally, the automatic recalibration of the generalist soft sensor was evaluated using two different bioprocesses: first, the biomass prediction of *Pichia pastoris* bioprocesses, and second, the biomass and protein prediction of *Bacillus subtilis* processes with variable process characteristics, such as cultivation media.

## 2. Materials and Methods

### 2.1. Pichia pastoris Process—Cultivation and Hardware

#### 2.1.1. Strain, Preculture Conditions, and Main Culture

The *P. pastoris* (DSMZ 70382) processes were performed at the Chair of Brewing and Beverage Technology (Technical University of Munich, Freising, Germany). As a preculture for the main process, three shake flasks (150 mL) were prepared with 50 mL of FM22 medium, and glycerol was added as a carbon source. The preculture was then cultured at 150 min^−1^ and 30 °C for 70 h. The FM22 medium contained the following: (NH_4_)_2_SO_4_, 5 g L^−1^; CaSO_4_ 2H_2_O, 1 g L^−1^; K_2_SO_4_, 14.3 g L^−1^; KH_2_PO_4_, 42.9 g L^−1^; MgSO_4_ 7H_2_O, 11.7 g L^−1^; and glycerol, 40 g L^−1^. To the FM22 medium, an additional 2 mL L^−1^ of the PTM4 solution was added: CuSO_4_ 5H_2_O, 2 g L^−1^; KI, 0.08 g L^−1^; MnSO_4_ H_2_O, 3 g L^−1^; Na_2_MoO_4_ 2H_2_O, 0.2 g L^−1^; H_3_BO_3_, 0.02 g L^−1^; CaSO_4_ 2H_2_O, 0.5 g L^−1^; CoCl_2_, 0.5 g L^−1^; ZnCl_2_, 7 g L^−1^; FeSO_4_ H2O, 22 g L^−1^; biotin, 0.2 g L^−1^; and conc. H_2_SO_4_, 1.0 mL. The media composition was taken from Stratton et al. [20].

The three preculture flasks were pooled and used as inoculum for the main cultivation (15 L working volume), also with FM22 and PTM4 solution as a medium. The primary cultivation was divided into three technical process phases: an initial batch phase using glycerol as a substrate, a transition phase without a substrate, and a fed-batch phase using methanol as a substrate. The methanol feed was supplemented with 12 mL L^−1^ of PTM4 solution. Temperature (30 °C), pressure (500 mbar), pH (5), and dissolved oxygen (40%) were all monitored and controlled throughout the process. A cascade control was used for dissolved oxygen control, which firstly adjusts the stirrer speed (300–600 min^−1^) and then the aeration rate (20–40 L min^−1^).

#### 2.1.2. Bioreactor, Sensor Systems, and Reference Measurements

The main cultivation was carried out in a Biostat^®^ Cplus bioreactor (42 L total volume, Sartorius AG, Goettingen, Germany). The reactor was equipped with standard pH, pressure, and dissolved oxygen sensors. Additionally, the methanol concentration was measured using an inline Alcosens sensor (Heinrich Frings GmbH & Co. KG, Rheinbach, Germany), and the CO_2_ and O_2_ concentrations in the exhaust gas were measured using a BlueInOne sensor (BlueSens gas sensors GmbH, Herten, Germany).

The reference measurements were made by sampling the process every 2 h with an autosampler. The biomass concentration in the samples was determined in triplicate using dry cell weight (DCW). Consequently, 2 mL of each solution was added to weighed centrifuge tubes and centrifuged at 21,000× *g*. The supernatant was then discarded, and the cell pellet was dried for three days at 80 °C before being weighed.

The above process parameters were controlled by the bioreactor’s controller unit. SIMATIC SIPAT (Siemens AG, Munich, Germany) was used for sensor, actuator, and reference value data recording.

### 2.2. Bacillus subtilis Process—Cultivation and Hardware

#### 2.2.1. Strain, Preculture Conditions, and Main Culture

The *B. subtilis* processes were performed at Clariant Produkte (Deutschland) GmbH (Planegg, Germany). A preculture cultivation strategy optimized by Clariant Produkte (Deutschland) GmbH was used to generate an inoculum for the main culture. The main culture (700 mL) was cultivated with two different media (CLA medium and FB medium). CLA medium represents an optimized high-performance medium for industrial cultivation, which is not described in detail due to confidentiality agreements. The second medium was the standard high-cell-density FB medium [21]. The FB medium was composed of the following components: K_2_HPO_4_, 4 g L^−1^; KH_2_PO_4_, 4 g L^−1^; Na_2_HPO_4_, 7 g L^−1^; (NH_4_)_2_SO_4_, 1.2 g L^−1^; NH_4_Cl, 0.2 g L^−1^; and peptone, 5 g L^−1^. Additionally, the following separately sterilized solutions were added to the medium: 2 mL L^−1^ MgSO_4_ solution (100 g L^−1^), 0.2 mL L^−1^ CaCl_2_ solution (40 g L^−1^), 4 mL L^−1^ glucose solution (500 g L^−1^), and 1 mL of a trace element solution (FeSO_4_, 8 g L^−1^; MnSO_4_, 2 g L^−1^; Na_2_MoO_4_, 0.4 g L^−1^; ZnSO_4_, 0.2 g L^−1^; AlCl_3_, 0.2 g L^−1^; CuCl_2_, 0.2 g L^−1^; H_3_BO_4_, 0.1 g L^−1^ all dissolved in 5 M HCl). The pH was increased during the process. Ammonium hydroxide and sulfuric acid were used for control. The temperature was reduced during the process. Glucose was used as the substrate, which was initially supplied for an initial batch phase and then fed later in the process (fed-batch phase). Oxygen was supplied to the process by the constant inflow (1.5 L min^−1^) of sterile air via a sparger.

#### 2.2.2. Bioreactor, Sensor Systems, and Reference Measurements

The processes were carried out in Multifors vessels (1.4 L total volume, Infors AG, Bottmingen, Switzerland). The vessels were outfitted with standard pH, pressure, and dissolved oxygen sensors. A mass spectrometer (Thermo Scientific™ Prima PRO, Thermo Fisher Scientific Inc., Waltham, MA, USA) was used to measure the exhaust gas inline.

Samples for the reference measurements were taken manually by trained laboratory personnel. Samples were then analyzed for biomass and protein concentration in triplicate. Biomass concentration was determined using colony forming units (CFU), as cell dry weight measurement was not applicable due to insoluble media components. To determine the CFU, 100 µL of the diluted sample was spread on LB plates and cultured at 37 °C for 2 days. Following that, the colonies formed were counted, and the CFU per mL were calculated. The target protein’s activity was measured to determine the protein concentration.

The data logging and process control were handled by the bioprocess platform software eve^®^ (Infors AG, Bottmingen, Switzerland).

For the graphical representation of the results using the generalist soft sensor approach in the *B. subtilis* process, protein, biomass, and time were converted to percent of the maximum value of the graphical representation due to confidentiality clauses.

### 2.3. Development of the Generalist Soft Sensor Concept

The soft sensor development and validation were performed in Python 3.8.2.

#### 2.3.1. Structure of the Soft Sensor Concept with Phase-Dependent Recalibration

**Figure 1:**
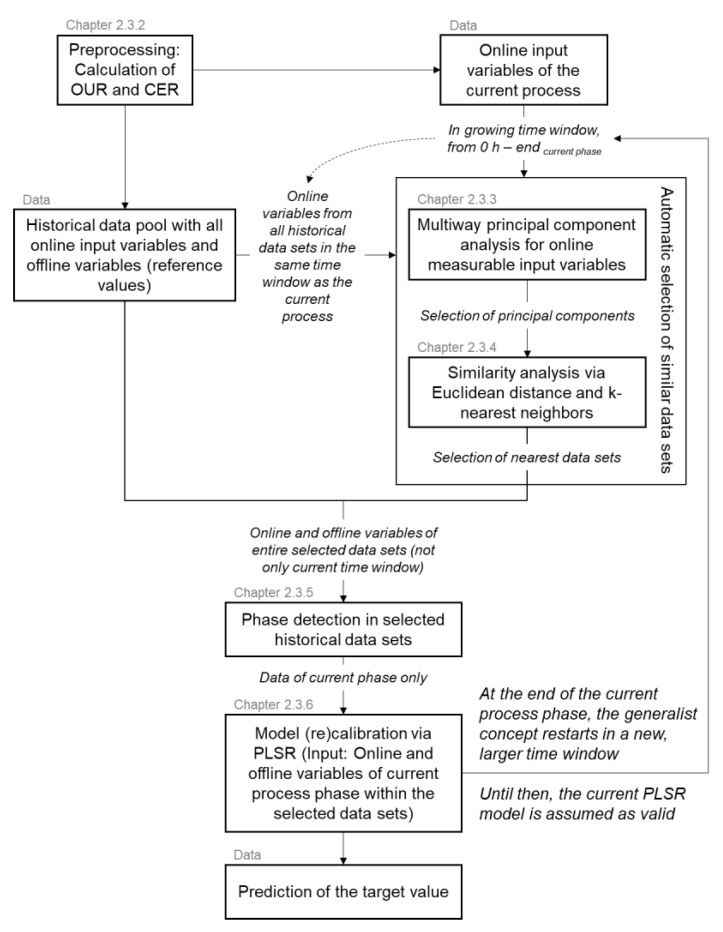
Structure of the generalist soft sensor concept. OUR = oxygen uptake rate; CER = carbon dioxide evolution rate; PLSR = partial least squares regression.

Figure 1 depicts the structure of the generalist soft sensor concept. For the evaluation, a data pool of 19 *P. pastoris* data sets and 72 *B. subtilis* data sets ($n_{CLA}=57,\;n_{FB}=15$) was available. Each data set represents a completed process. The algorithm was designed for real-time application during a process. For the validation of the algorithm, a single historical data set is always selected as the current process. The remaining historical data sets of an organism are assigned to the historical data pool. The algorithm is given the validation data set as if it was currently taking place, meaning that the data set is not completely transferred to the algorithm at the start, but grows over time. The first calibration is performed after 1 h of process time to initialize the generalist soft sensor concept. The algorithm uses existing hardware sensors, actuators, and additionally calculated variables as input. Only the calibration of the soft sensor model by means of PLSR accesses the offline determined reference values of the target variable from the automatically selected historical data sets. The following chapters describe the main intermediate steps of the generalist soft sensor concept.

#### 2.3.2. Preprocessing of the Data Sets

At first, additional input variables, such as the OUR and the CER, were calculated. This required variables such as the airflow rate ${\dot{V}}_{air}$, pressure p, the liquid reactor volume $V_{liquid}$, the universal gas constant R ($8.314\;\cdot \;10^{-2}\frac{L\;bar}{mol\;K}$), the temperature T, and the mole fractions of oxygen $x_{O2}$ and carbon dioxide $x_{CO2}$ in the inlet (index in) and outlet (index out) [22].

(1) $$\mathrm{CER}=\frac{{\dot{V}}_{air}\cdot p}{V_{liquid}\cdot R\cdot T}\cdot \left(\frac{1-x_{O2,\;in}-x_{\mathrm{CO}2,in}}{1-x_{O2,out}-x_{\mathrm{CO}2,out}}\cdot x_{\mathrm{CO}2,out}-x_{\mathrm{CO}2,in}\right)\;$$

(2) $$\mathrm{OUR}=\frac{{\dot{V}}_{air}\cdot p}{V_{liquid}\cdot R\cdot T}\cdot \left(x_{O2,in}-\frac{1-x_{O2,\;in}-x_{\mathrm{CO}2,in}}{1-x_{O2,out}-x_{\mathrm{CO}2,out}}\cdot x_{O2,out}\right)$$

Most of the variables needed for the calculation were measured directly with hardware sensors. Only the liquid reactor volume $V_{liquid}$ was calculated by balancing the start volume, liquids added during the process (pH correction agent, antifoam, substrate feed), and liquids removed from the process (samples). Evaporation was neglected in the balance.

As input to the generalist soft sensor concept, the CER and OUR were used as rates as well as cumulative values.

#### 2.3.3. Multiway Principal Component Analysis

After preprocessing, an MPCA was performed for the current time span with the online available input variables of the data pool. Using an MPCA offers the benefit of applying an ordinary PCA to a three-dimensional matrix. During the MPCA, the data pool was first refolded. Then, the three dimensions of the data pool (batch I, online variables J, and time K) were folded batchwise, implying that the I×J×K data matrix becomes an I×JK matrix [11]. Subsequently, a principal component analysis was performed on the refolded data matrix, the principal components explaining 95 % of the total variance were selected, and the corresponding scores were passed to the following similarity analysis.

#### 2.3.4. Similarity Analysis via Euclidean Distance and k-Nearest Neighbors

The similarities between the data pool and the current data set were determined using the calculated scores. Consequently, the Euclidean distance dk of the scores between the current process $t_{current}$ and all historical processes $t_{historical}$ was computed for all principal components A employed.

(3) $$dk=∥t_{current}-t_{historical}∥=\sqrt{\overset{A}{\underset{i=1}{\sum }}{\left(t_{current,\;i}-t_{historical,i}\right)}^{2}}$$

Then, based on the Euclidean distance, the k-nearest neighbors were selected. $k_{neighbors}$ could be defined depending on the size of the data pool, the sampling frequency, or on a limit value for Euclidean distance dk.

#### 2.3.5. Phase Detection

The process phases in the selected k historical data sets were then determined using a three-stage data-driven phase detection method. Burrs, or faults in phase detection, were also filtered by this algorithm. Lu et al. [16] and Wang et al. [18] inspired the phase detection algorithm.

**Figure 2:**
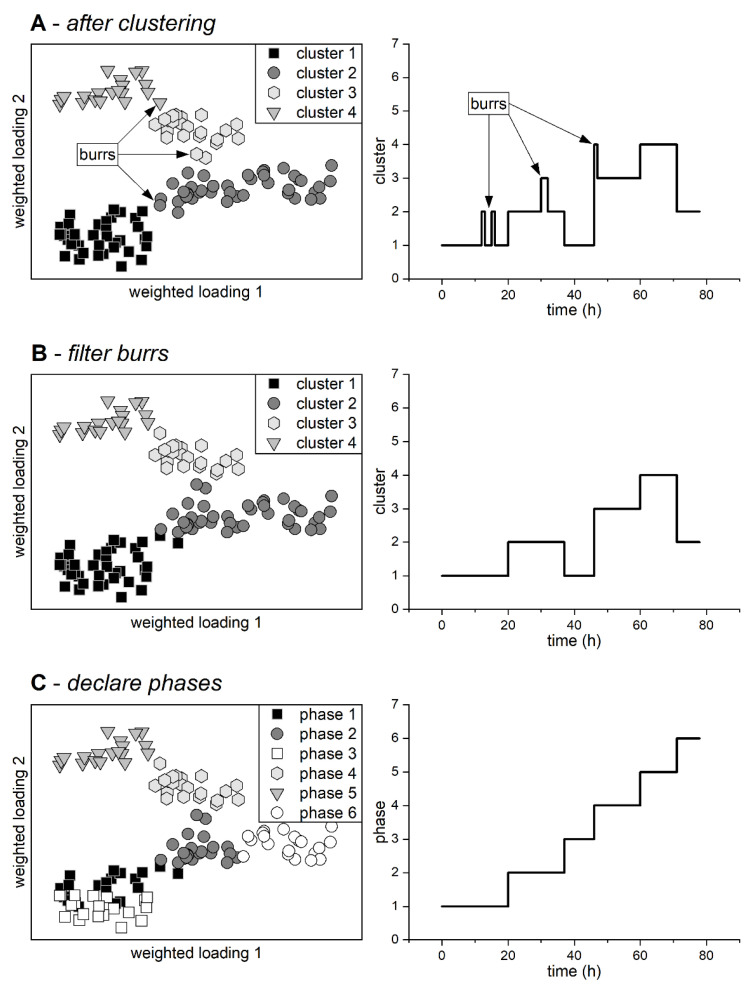
Various stages of generalist phase detection. The clustering of time segments within processes is shown schematically on the left, based on the weighted loadings of the principal component analysis. Because the actual loadings would represent a high-dimensional space, the representation is schematic. The temporal occurrence of similarity clusters within the selected historical processes is shown on the right. (**A**) Principal component analysis and *k*-means clustering are used to detect raw similarity clusters. (**B**) The burrs are filtered (outliers in clustering caused by sensor faults). (**C**) Using the clusters’ temporal occurrence to declare phases.

The chosen data sets were firstly divided into 1 h segments. Each of these segments was then subjected to principal component analysis. Eigenvalue-weighted loadings, which describe the correlations between the online process variables for each time segment, could then be calculated for each principal component analysis. k-means clustering was used to identify similarity clusters in this eigenvalue-weighted loadings space. The number of technical process phases was used as $k_{cluster}$ here. As in the eigenvalue-weighted loadings space, the detected similarity clusters could then be displayed in chronological order (see Figure 2A). Sensor failures frequently result in faulty loading matrices, which then leads to phase detection outliers (burrs), as illustrated in Figure 2A. For the detection and correction of burrs, the chronological order of cluster assignment of time segments r per time step i was utilized. Furthermore, the coordinates of the cluster centroids *m* and the individual time segments *v* in eigenvalue-weighted loadings space were used.

(4) $$burr_{i}\{\begin{array}{r} r_{i}\neq r_{i+1}\;\wedge \;r_{i+1}=r_{i-1\;\;}\;\;:\;r_{i,new}=r_{i+1}\\ r_{i}\neq r_{i+1}\wedge \;r_{i}\neq r_{i-1}\wedge \;r_{i+1}\neq r_{i-1}\wedge \left|v_{r_{i}}-m_{r_{i+1}}\right|\leq \left|v_{r_{i}}-m_{r_{i-1}}\right|\;\;\;:\;r_{i,new}=r_{i+1}\\ r_{i}\neq r_{i+1}\wedge \;r_{i}\neq r_{i-1}\wedge \;r_{i+1}\neq r_{i-1}\wedge \left|v_{r_{i}}-m_{r_{i+1}}\right|>\left|v_{r_{i}}-m_{r_{i-1}}\right|\;\;\;:\;r_{i,new}=r_{i-1} \end{array}$$

To detect and correct burrs that are longer than one time segment, successive time segments of the same cluster can be combined into a single time segment. Subsequently, this time segment can be corrected under the same conditions as in Formula 4. The maximum length of the combined time segments *η* can be chosen based on the total process length. For this study, it was set to two successive time segments. Figure 2B depicts the filtered clusters. Finally, the process phases could be identified. Consequently, the clusters were classified into several phases based on their temporal occurrence (see Figure 2C).

More process phases were created than were originally specified by $k_{cluster}$. However, this can result in more accurate phase detection, particularly for processes where the exact number of phases is unknown (due to other phases besides the technical phases, such as biological phases caused by, e.g., oxygen limitation).

#### 2.3.6. Partial Least Squares Regression

Following phase detection, the current process could be matched with the detected phases of the selected historical data sets to determine the current process phase. The hardware sensor readings, actuators, and reference values from the selected historical data sets from this period were then used to generate a temporary soft sensor model which was valid until the end of the current phase. The algorithm then returned to 2.3.3 and resumed with a multiway principal component analysis to select similar data sets.

A linear model structure was used for the soft sensor predictions $\hat{y}$ of the generalist concept. Therefore, the process variables matrix X and the parameters b calculated through PLSR were used.

(5) $$\hat{y}=Xb$$

PLSR is widely used in bioprocesses [2]. With the calibration of the prediction model, this methodology implements the target variable-based dimensionality reduction in the input variables. Consequently, latent variables are chosen based on the target variable’s explained variance. For this, the selected historical data sets were iterated as calibration and validation data. This allowed for cross-validation and avoided overfitting. The prediction performance of the models was calculated as a function of the number of latent variables j by the mean squared error (MSE). Therefore, the respective model $\hat{y}$ predictions as well as the n reference values $y_{hist}$ of the historical data sets were used.

(6) $${\mathrm{MSE}}_{j}=\frac{1}{n}\overset{n}{\underset{i=1}{\sum }}{\left(y_{hist}-{\hat{y}}_{j}\right)}^{2}$$

The optimal number of latent variables was determined based on the first local minimum of the MSE.

#### 2.3.7. Quality Parameters for Evaluation of the Generalist Soft Sensor Concept

Quality parameters such as the root mean squared error of prediction (RMSEP) and relative error were calculated to evaluate the generalist soft sensor concept. The validation data set’s reference values $y_{ref}$, the generalist soft sensor $\hat{y}$, and the maximum value of the target variable $y_{max}$, as well as the target variable’s minimum value $y_{min}$, were, thus, employed.

(7) $$\mathrm{RMSEP}=\sqrt{\frac{1}{n}\overset{n}{\underset{i=1}{\sum }}{\left(y_{ref}-\hat{y}\right)}^{2}}$$

(8) $$relative\;error=\frac{RMSEP}{y_{max}-y_{min}}$$

## 3. Results and Discussion

**Figure 3:**
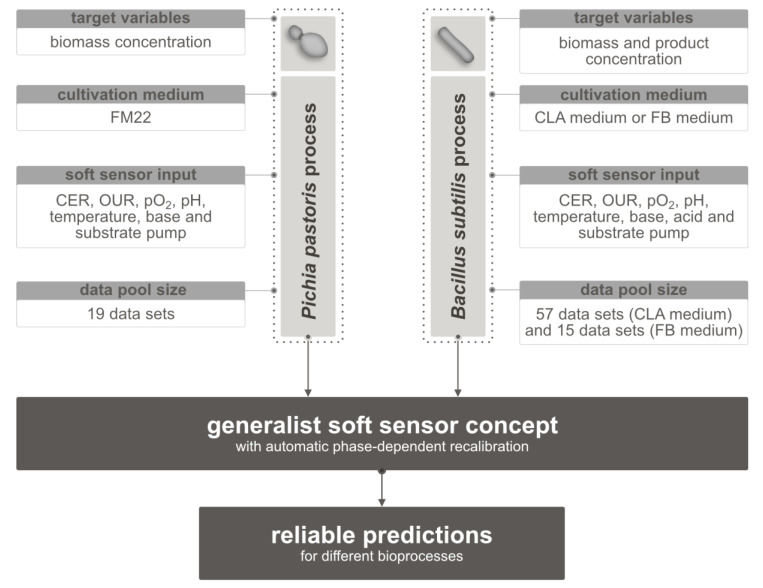
Overview of the different target variables, media, soft sensor input, and the data pool sizes of the bioprocesses. CER = carbon dioxide evolution rate; OUR = oxygen uptake rate.

The following is the structure of the evaluation of the generalist soft sensor concept. First, the algorithm’s function was validated on the *P. pastoris* process. The temporal change in the Euclidean distances of the historical data pool to the current process is shown and discussed, as is the course of the predictions with detected phases of the example process. Following that, the validation of the *B. subtilis* process is demonstrated, particularly the differentiation of the different media in the automated selection of data sets. On an example data set, the profile of the predictions and phase detection is also shown. Finally, the relative errors of the various applications of the generalist soft sensor concept are compared. Figure 3 depicts an overview of the various process characteristics of the bioprocesses.

### 3.1. Evaluation of the Generalist Soft Sensor on the P. pastoris Process

**Figure 4:**
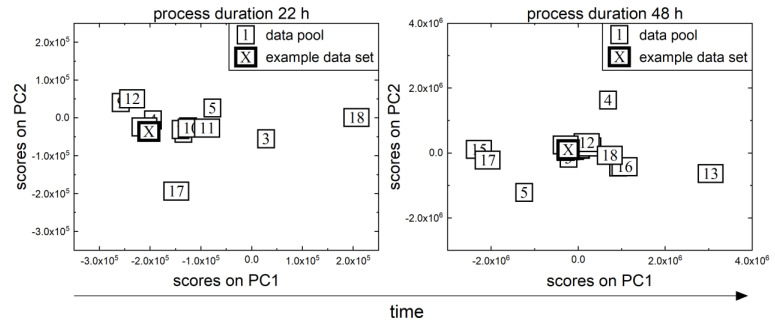
The MPCA of the *P. pastoris* data pool at two different time points is depicted (22 and 48 h). Each box represents a different data set; the number indicates the batch number. The results of the first two principal components of the data pool (thin frame) and the example data set are shown (thick frame). PC = principal component.

Figure 4 depicts the selection of historical data sets from the *P. pastoris* data pool (19 data sets) for the *P. pastoris* example process. Two distinct time points were chosen: 22 h for the start of the growth batch phase and 48 h for the start of the fed-batch phase. The data pool’s spatial distribution shifts over time. Thus, the generalist soft sensor concept selected data sets 4, 6, 7, and 12 at 22 h and data sets 1, 6, 9, and 14 at 48 h, similar to the validation data set. This demonstrates that even within a process, the most similar data sets can change because as the process progresses, more and more information on the current process is available, allowing a more appropriate selection of data sets. First, the current process is compared with historical data sets in a growing time window in the generalist soft sensor concept described in this study (*start process* to *current process time*). Following that, phases are determined in this growing time window, and a flexible phase-dependent recalibration time window is calculated, in which the data points of the selected historical data sets are used to calibrate the currently valid soft sensor model.

**Figure 5:**
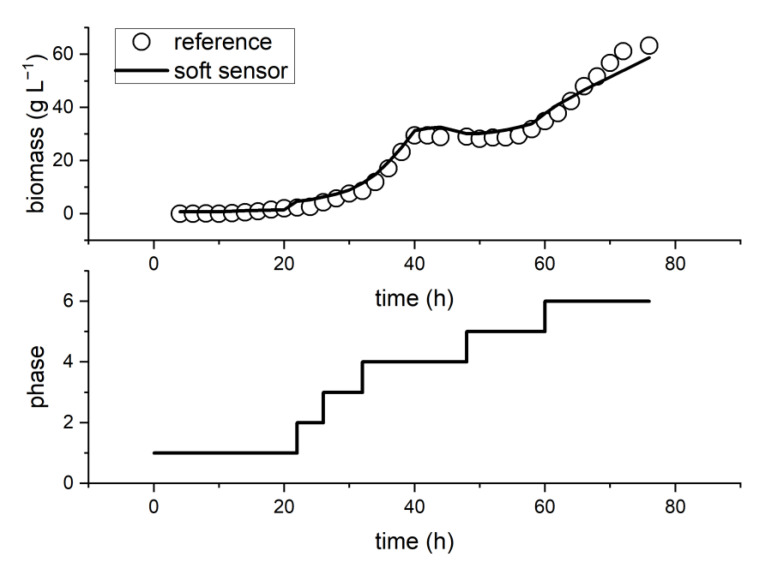
Prediction of the generalist soft sensor concept with reference values and detected phases for the biomass prediction of an example data set of the *P. pastoris* process.

Figure 5 depicts the temporal evolution of biomass concentration with the prediction from the generalist soft sensor concept, as well as reference values. A high prediction performance can be seen. Only in the last process phase does the prediction performance deteriorate. One reason could be that several of the selected calibration data sets terminated early. As a result, fewer calibration data points were available for the soft sensor model in this phase, which could lead to a decrease in the prediction performance. Quality parameters such as RMSEP (2.6 g L^−1^) and relative error (4.1%) were within acceptable limits (relative error < 10%).

Six distinct phases were detected through automatic phase detection. The phases can be classified as follows: Phase 1 is the lag phase; Phase 2 is the start of the growth batch phase (pO_2_ = 100%); Phase 3 is the beginning of the stronger growth batch phase (significant decrease in pO_2_); Phase 4 is the main batch phase and transition phase (pO_2_ controlled at 40% until substrate reaches 0 g L^−1^); Phase 5 is the adaptation to a new substrate and the start of the fed-batch phase; and Phase 6 is the completion of adaptation to the new medium and the second part of the fed-batch phase. The detected phases can, in theory, be justified both technically and biologically. The division of the batch phase into three phases can be attributed primarily to differences in oxygen saturation in the medium, as well as the start of control thereof. However, because the historical data sets had a high sample frequency (2 h), these shorter phases did not pose challenges. If the historical data sets had a lower sampling frequency, the number of data sets to be selected would have to be increased to have enough reference points available for short phases.

### 3.2. Evaluation of the Generalist Soft Sensor Concept on a B. subtilis Process with Changing Process Characteristics

**Figure 6:**
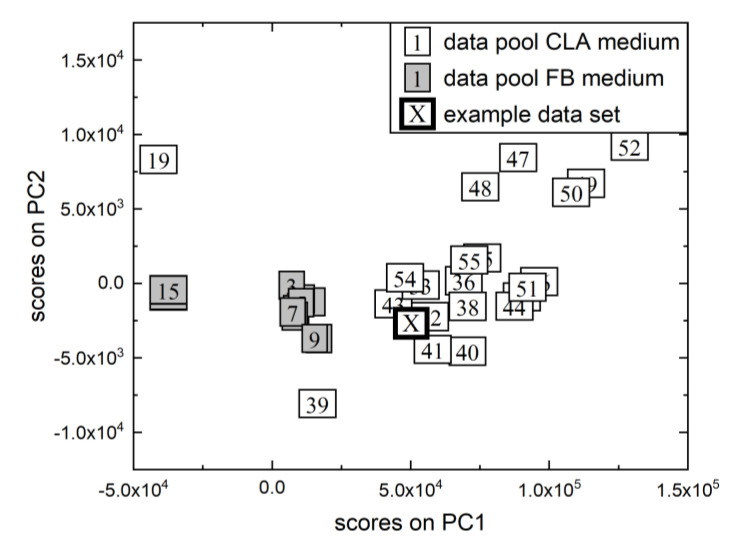
Illustration of the MPCA of the *B. subtilis* data pool with different media. Each box represents a different data set; the number indicates the batch number. The scores of the data pool’s first two principal components (thin frame and white filling: CLA medium, thin frame and gray filling: FB medium) and the example data set are shown (thick frame). PC = principal component.

The generalist soft sensor concept was then put to the test with an example data set from the *B. subtilis* process. Figure 6 depicts the outcomes of the selection of similar historical data sets from the *B. subtilis* data pool ($72\;\text{data}\;\mathrm{sets}\;\mathrm{with}\;n_{CLA}=57\;\mathrm{and}\;n_{FB}=15)$. Because the sampling frequency for *B. subtilis* was lower and the data pool was larger than that for *P. pastoris*, five similar data sets were always selected instead of four. Even during short phases, there should be enough reference points for calibration. The data pool included both data sets with CLA medium and FB medium. Visually, the separability of the various process characteristics can be confirmed. This implies that the existing online hardware sensors used as input into the generalist soft sensor concept provided enough information about the process to reflect differences in media compositions and their impact on process progress.

**Figure 7:**
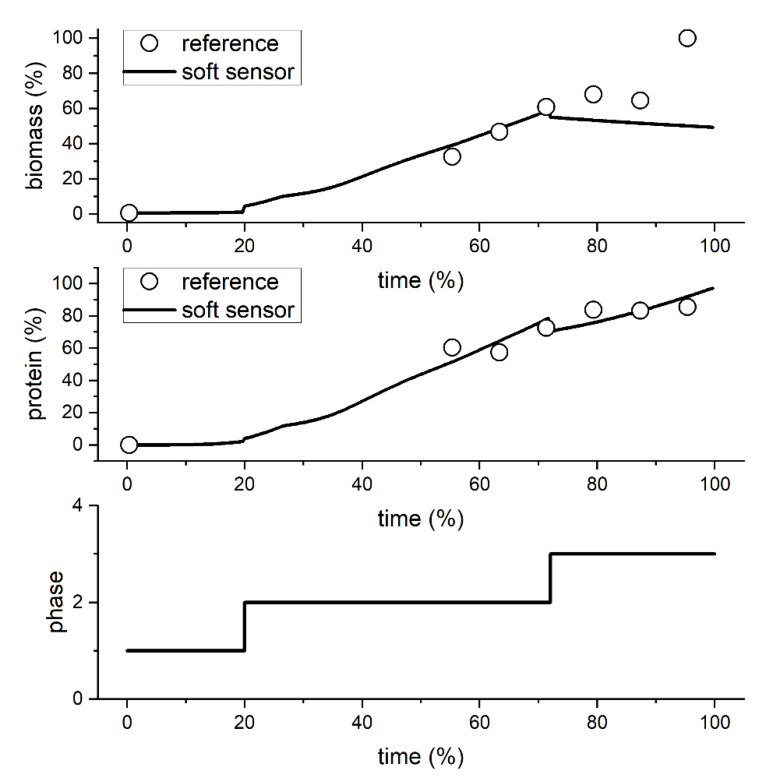
Prediction of the generalist soft sensor concept with reference values and detected phases for the biomass and protein prediction of an example data set of the *B. subtilis* process with CLA medium. Axes are in % due to confidentiality agreements.

The predictions of the *B. subtilis* example data set were achieved with relative errors of 20.4% (biomass prediction) and 7.2% (protein prediction) using the generalist soft sensor concept. At the last reference point of the biomass concentration, an untypically high CFU was measured, which indicates an outlier. The relative error of the biomass prediction without this outlier is 13.2%. Figure 7 shows a visual confirmation of the high prediction performance. The algorithm identified three distinct phases, which are as follows: Phase 1: Batch phase (oxygen saturation drops to 0%); Phase 2: Start of fed-batch phase (oxygen saturation rises again due to substrate limitation); Phase 3: Second part of fed-batch phase (oxygen saturation returns to a stable, high level). Thus, the detected phases can be technically and biologically assigned and are, therefore, valid. The validation example here was only for predictions in CLA medium, but the applications of the generalist soft sensor concept for the *B. subtilis* process in FB medium are discussed in the following section. In general, the *B. subtilis* process has already confirmed the successful use of the generalist soft sensor concept for the more industrially relevant target variable product concentration.

### 3.3. Overall Evaluation of the Generalist Soft Sensor Concept

Finally, the generalist soft sensor concept’s prediction performance for all use cases was validated. Five random validation data sets were chosen for each use case, and the average relative error with standard deviation was calculated and summarized in Table 1. For comparison, a concept with fixed time windows was used as reference. Therefore, the algorithm of the generalist concept was modified with a fixed window size of 20 h (ensuring a sufficient amount of calibration points for all use cases) instead of dynamic phase-dependent windows.

Comparing the predictions of the generalist concept with the reference concept, a significantly better prediction performance can be observed for all use cases. This demonstrates that, especially for multiphase bioprocesses, an automated phase detection and a subsequent dynamic adaptation of the windows to the phases are essential for a suitable prediction performance. Particularly when there are fewer reference points for the target variable in the calibration data, phase-dependent allocation is important, as can be seen when comparing the prediction performance of the *P. pastoris* and *B. subtilis* process models with the fixed window concept.

The generalist concept has a similar prediction performance for biomass prediction in the *P. pastoris* process and product prediction in the *B. subtilis* process. Comparing the biomass prediction results for the *P. pastoris* process of the generalist soft sensor with the hybrid soft sensor model of Brunner et al. [3], a model without similarity analysis and selection of similar data sets, but with knowledge-based phase detection and process knowledge in terms of a carbon balance, an approximately comparable prediction performance could be achieved ($relative\;error_{Brunner\;et\;al.}=5.5\;\%$). The prediction performance for biomass concentration of the *B. subtilis* process is lower than the predictions of the other target variables. However, the primary reason for the lower prediction performance is not the generalist soft sensor concept itself, but the higher measurement error of the biomass reference measurement during the *B. subtilis* process $\left(relative\;measurement\;error_{CFU}\approx 7–30\%\right)$ than the protein reference measurement $\left(relative\;measurement\;error_{activity}\approx 0.8\%\right)$ and the biomass reference measurement during the *P. pastoris* process $\left(relative\;measurement\;error_{DCW}\approx 0.7\%\right)$. Particularly in the CFU measurement, the necessary high dilutions of the samples led to an absolute error that increases with the level of dilution. Comparing the prediction performance for the same target variable in different media in the *B. subtilis* process, similar high relative errors could be observed. Thus, it can be confirmed that the differences in the prediction performance between the different targets can be predominantly attributed to the different measurement errors of the reference measurements.

Consequently, it was possible to demonstrate that the generalist soft sensor concept is suitable for predicting different scenarios, even when process characteristics such as media, strains, and target variables are varied.

## 4. Conclusions

This study revealed that a generalist soft sensor concept could reliably predict target variables in bioprocesses with varying process characteristics. The biomass prediction for the *P. pastoris* process and the biomass and product prediction for the *B. subtilis* process were utilized to evaluate this concept.

Since the generalist concept is real-time capable, it can be used for process monitoring as well as for process control. For process monitoring, the predicted variables are used and expected process corridors can additionally be created for them to be able to directly assess the quality of the process. For process control, the predictions can be implemented directly in a control concept. However, it is recommended to add a smoother phase transition in the generalist concept for this application. As well as biomass and product concentration, additional target variables such as substrate concentration could be predicted with the generalist concept, enabling further control strategies. The major challenge in applying the generalist soft sensor concept is gathering enough process information to digitally map the process, for example using hardware sensors. The concept is designed in such a way that hardware sensors, actuators, and additionally calculated variables other than those used in this study can also be used as the input variables. Additionally, if non-information-bearing input variables are present, the generalist concept will automatically give them very little or no influence on the prediction model. However, if the existing online input variables generally do not contain enough information about the process, reliable predictions cannot be made, even with the generalist concept. Furthermore, as large a data pool as possible should be provided because relevant prediction models can only be trained if current process variations have already been recorded in similar historical data sets. The following topics can be considered for future application and further development of the proposed generalist soft sensor concept. One optimization possibility is automatic data pool maintenance. For example, previous data sets based on online process variables may be similar to the current process but have faulty reference values. This can occur due to incorrect sampling or measurement issues with the samples. To overcome this, an automated concept that removes outliers during data pool preprocessing can be implemented. One implementation approach is to group similar data sets based on their online variables, as presented in this study, but then, the correlations between the reference and online variables of the historical data sets are compared. Individual data sets with significantly differing correlations can be removed. Another optimization possibility is the addition of a synchronization method [23] to prepare data sets with varying lengths for MSPC-based selection because previous data sets that indicate similar temporal profiles of the online variables to the current process are chosen for automatic recalibration. However, this neglects the fact that data sets may be adequate for recalibration despite their temporal variances.

This concept can be tested on other multiphase bioprocesses in the future to overcome isolated solutions in soft sensor applications and proceed toward soft sensor concepts comprising various bioprocesses.

## Figures and Tables

**Table 1:** Comparison of the relative root mean square errors of the generalist soft sensor concept and the reference concept with fixed window size of 20 h for biomass prediction of the *P. pastoris* process and the biomass and product prediction of a *B. subtilis* process in two different cultivation media. Five random data sets each were used for validation. DCW = dry cell weight; CFU = colony forming units.

			Relative Error of Prediction in %	
Organism	Medium	Target Variable	Generalist Soft Sensor Concept	Reference Concept with Fixed Window Size
*P. pastoris*	FM22	biomass (DCW)	6.9 ± 3.4	9.8 ± 4.3
*B. subtilis*	CLA	biomass (CFU)	12.2 ± 5.1	60.1 ± 37.7
*B. subtilis*	CLA	protein (activity)	7.2 ± 2.3	34.3 ± 15.2
*B. subtilis*	FB	biomass (CFU)	12.8 ± 3.9	54.6 ± 7.6
*B. subtilis*	FB	protein (activity)	8.8 ± 2.0	49.4 ± 39.0

## Data Availability

The *P. pastoris* data sets that support the findings of this study are available from the corresponding author upon reasonable request. *B. subtilis* data sets cannot be shared due to confidentiality agreements.
